# Bacteriophage EK99P-1 alleviates enterotoxigenic *Escherichia coli* K99-induced barrier dysfunction and inflammation

**DOI:** 10.1038/s41598-022-04861-4

**Published:** 2022-01-18

**Authors:** Narae Kim, Min Jeong Gu, Yoon-Chul Kye, Young-Jun Ju, Rira Hong, Do Bin Ju, Young Jin Pyung, Seung Hyun Han, Byung-Chul Park, Cheol-Heui Yun

**Affiliations:** 1 Department of Agricultural Biotechnology, and Research Institute of Agriculture and Life Sciences, Seoul National University, Seoul, 08826 Republic of Korea; 2 Department of Oral Microbiology and Immunology, and Dental Research Institute, School of Dentistry, Seoul National University, Seoul, 08826 Republic of Korea; 3 Institutes of Green-Bio Science Technology, and Graduate School of International Agricultural Technology, Seoul National University, Pyeongchang, 25354 Republic of Korea; 4 Center for Food and Bioconvergence, Seoul National University, Seoul, 08826 Republic of Korea

**Keywords:** Immunology, Microbiology

## Abstract

Bacteriophages, simply phages, have long been used as a potential alternative to antibiotics for livestock due to their ability to specifically kill enterotoxigenic *Escherichia coli* (ETEC), which is a major cause of diarrhea in piglets. However, the control of ETEC infection by phages within intestinal epithelial cells, and their relationship with host immune responses, remain poorly understood. In this study, we evaluated the effect of phage EK99P-1 against ETEC K99-infected porcine intestinal epithelial cell line (IPEC-J2). Phage EK99P-1 prevented ETEC K99-induced barrier disruption by attenuating the increased permeability mediated by the loss of tight junction proteins such as zonula occludens-1 (ZO-1), occludin, and claudin-3. ETEC K99-induced inflammatory responses, such as interleukin (IL)-8 secretion, were decreased by treatment with phage EK99P-1. We used a IPEC-J2/peripheral blood mononuclear cell (PBMC) transwell co-culture system to investigate whether the modulation of barrier disruption and chemokine secretion by phage EK99P-1 in ETEC K99-infected IPEC-J2 would influence immune cells at the site of basolateral. The results showed that phage EK99P-1 reduced the mRNA expression of ETEC K99-induced pro-inflammatory cytokines, *IL-1β* and *IL-8*, from PBMC collected on the basolateral side. Together, these results suggest that phage EK99P-1 prevented ETEC K99-induced barrier dysfunction in IPEC-J2 and alleviated inflammation caused by ETEC K99 infection. Reinforcement of the intestinal barrier, such as regulation of permeability and cytokines, by phage EK99P-1 also modulates the immune cell inflammatory response.

## Introduction

Enterotoxigenic *Escherichia coli* (ETEC)-associated diarrhea is an economically important disease^1,2^ that causes high morbidity and mortality in neonatal and weaned piglets^3,4^. Most, if not all, ETECs adhere to small intestinal epithelial cells (IECs) via fimbriae-mediated adhesion^5^, as an initial step in infection^6^. Once they enter the host cell, enterotoxins like heat-labile toxin (LT) or heat-stable toxin (ST) are secreted, increasing the paracellular permeability of the host^7,8^. Porcine intestinal epithelial cell line (IPEC-J2), originating from the small intestine jejunum of pigs, is used as a model for studying interactions between intestinal cells and enteric bacteria, including ETEC^9^.

Previous studies have shown that ETEC-infected IPEC-J2 exhibit decreased expression of tight junction proteins such as occludin, claudin, and zonula occludens-1 (ZO-1), coincident with the secretion of chemokines such as monocyte chemoattractant protein (MCP)-1 and interleukin (IL)-8, which recruit immune cells into the local inflammatory site^10^. Pro-inflammatory cytokines, including IL-1β, IL-2, IL-6, IL-12p40, interferon (IFN)-γ, and tumor necrosis factor (TNF)-α, have been observed in sera from ETEC-infected pigs^11^.

Diarrheal diseases caused by ETEC in the pig industry can be prevented and treated using antibiotics^12,13^; however, concerns over the increasing emergence of antibiotic-resistant bacteria together with global bans of antibiotics as feed additives have prompted efforts to develop alternatives to antibiotics^14^, including lytic bacteriophages and their lysines.

Bacteriophages simply phages have long been used as an alternative to antibiotics to treat bacterial infection^15^. Phage-in-feed have been shown to reduce the severity of ETEC-induced diarrhea in newborn^16^ and post-weaning^17,18^ piglets; they have also been shown to alleviate symptoms such as increased rectal temperature and *E. coli* adhesion concordant with reducing serum IL-8 and TNF-α^12^. However, limited information is available on the effect of phages on IECs in terms of regulating host immune responses upon ETEC infection.

Therefore, in this study, we investigated the beneficial effects of phage EK99P-1 on barrier protection in IECs, and the inflammatory response involving mucosal immune cells, under ETEC K99 infection.

## Results

### Phage EK99P-1 restrained intestinal barrier disruption

**Figure 1:**
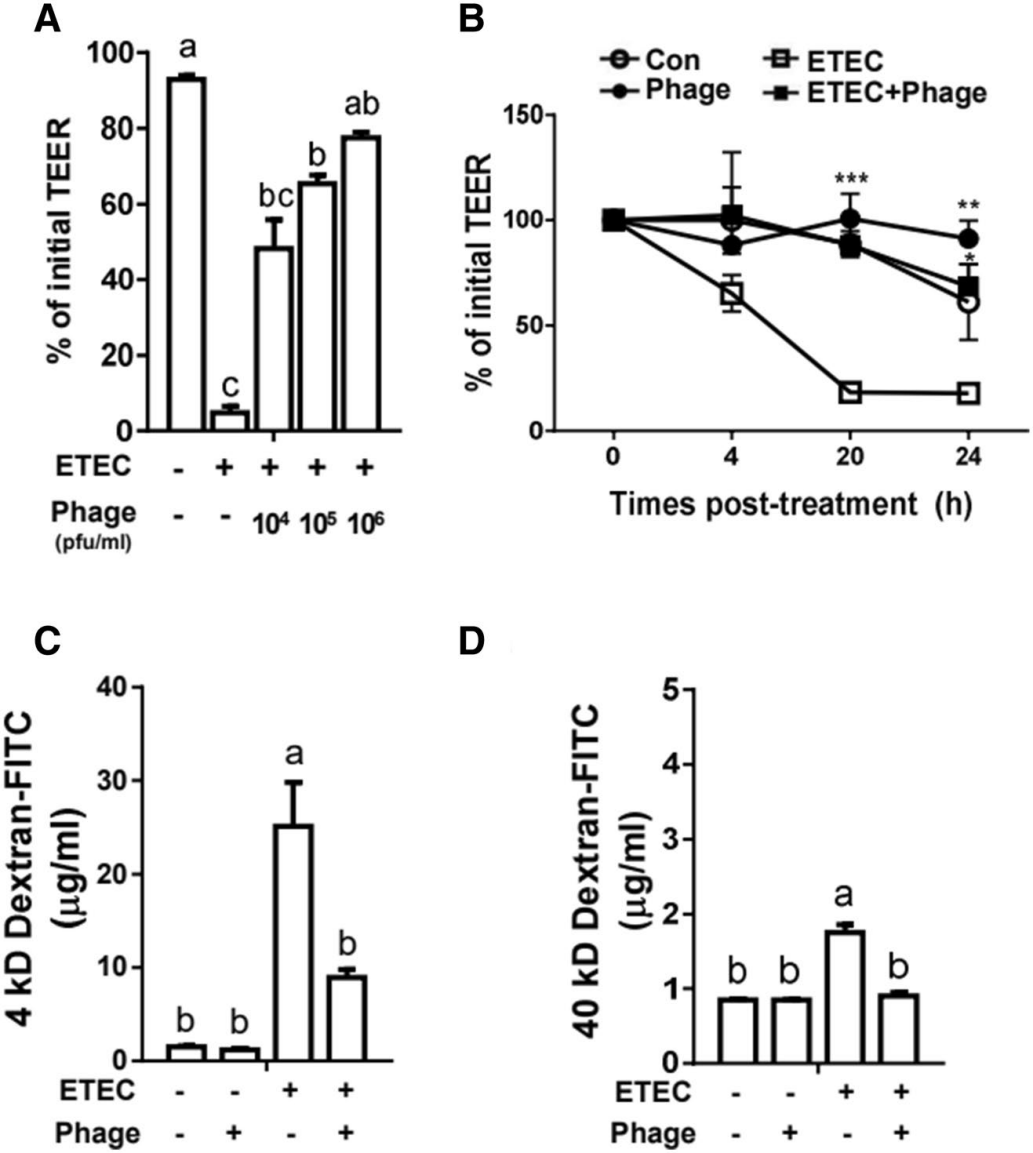
Phage EK99P-1 restored intestinal permeability in porcine intestinal epithelial cell line IPEC-J2 infected with enterotoxigenic *Escherichia coli* (ETEC K99). Differentiated IPEC-J2 were treated with ETEC K99 (1 × 10^7^ cfu/mL) and phage EK99P-1 (1 × 10^6^ pfu/mL) for 24 h. (**A**) Transepithelial electrical resistance (TEER) was measured in epithelial volt/ohm after 24 h of infection and (**B**) at the indicated time points. Each datum represents a percentage of initial TEER (n = 3). *P <0.05; **P < 0.01; ***P < 0.001. (**C,D**) Permeability was assessed by measuring 4- or 40-kD fluorescein isothiocyanate (FITC)-dextran transport after 24 h of infection (n = 3). Data are expressed as means ± standard deviation (SD). Means were compared by one-way analysis of variance (ANOVA), followed by the Friedman test and Tukey’s multiple comparison test. Different letters in each group indicate significant differences at *P* < 0.05.

ETEC induces increased intestinal permeability, resulting in reduced barrier protection^8^. To determine whether phage EK99P-1 treatment alleviates intestinal barrier function damage caused by ETEC K99 infection, we treated differentiated IPEC-J2 with either ETEC K99 or phage EK99P-1, or both, on the apical side. Decreased transepithelial electrical resistance (TEER) values due to ETEC K99 infection were restored by phage EK99P-1 treatment in a dose-dependent manner (Fig. 1A). IPEC-J2 cell death did not occur at any concentration of phage EK99P-1 in this experiment (Fig. S1). TEER values of differentiated IPEC-J2 were maintained until 24 h after treatment with both ETEC K99 and phage EK99P-1, whereas treatment with ETEC K99 alone significantly reduced TEER (Fig. 1B). This result suggests that phage EK99P-1 treatment minimizes damage and inhibits the increased permeability induced by ETEC K99.

Soluble toxins, such as LT and ST, are able to permeate the IEC barrier. The molecular weights of LT and ST are ~ 80 and 2 kD, respectively; they may migrate to the lamina propria across the epithelial layer according to their paracellular permeability^2^. To test this hypothesis, we used a dextran-FITC trans-epithelial permeability assay to quantify the effect of ETEC K99 and phage EK99P-1 on paracellular permeability: 4- or 40-kD dextran-FITC permeability was maintained in IPEC-J2 treated with ETEC K99 and phage EK99P-1, whereas ETEC K99 alone showed a significantly increased dextran permeability (Fig. 1C,D). These results demonstrate that phage EK99P-1 efficiently prevented the increased intestinal permeability associated with ETEC K99 infection. No significant changes in permeability were observed upon treatment with phage EK99P-1 alone, because no target bacteria of phage EK99P-1 were present (Fig. 1C,D). Taken together, these results show that phage EK99P-1 ameliorates the intestinal barrier disruption and permeability associated with ETEC K99 infection.

### Phage EK99P-1 inhibited the loss of tight junction integrity in IPEC-J2 infected with ETEC K99

**Figure 2:**
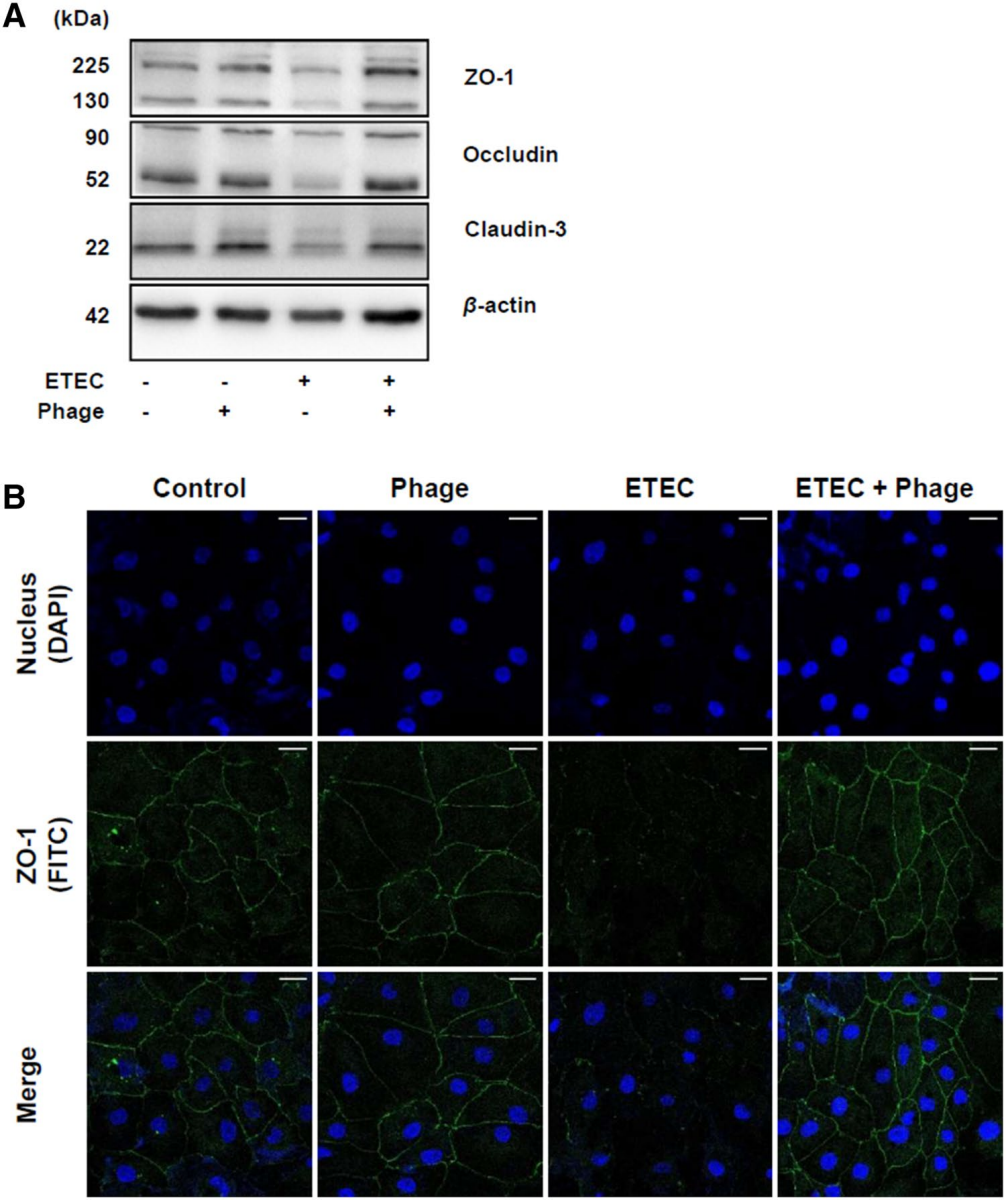
Phage EK99P-1 restored the decreased expression of tight junction proteins by ETEC in IPEC-J2 infected with ETEC K99. A monolayer of confluent IPEC-J2 was treated with ETEC K99 (2 × 10^6^ cfu/mL) and phage EK99P-1 (2 × 10^5^ pfu/mL) for 3 h. (**A**) Whole-cell lysates were analyzed for the expression of ZO-1, occludin, claudin-3, and *β*-actin using anti-claudin-3, -occludin, -ZO-1 and -β-actin antibodies by western blot assay (n = 3). Full-length blots/gels are presented in Supplementary Fig. 4. (**B**) ZO-1 expression in IPEC-J2 was visualized using confocal microscopy after staining with anti-ZO-1 antibody conjugated with Alexa Fluor 488-FITC (green) and nuclei with DAPI (blue) (n = 3). Scale bar = 30 μm.

Our previous study showed that differentiated IPEC-J2 form a polarized monolayer with increased TEER values, concordant with increased expression of tight junction proteins such as ZO-1, occludin, and claudin-3^19^. Because the increase in permeability is partly caused by reduced expression of these tight junction proteins, we examined the expression of ZO-1, occludin, and claudin-3 in IPEC-J2 infected with ETEC K99. Consistent with our permeability results (Fig. 1), disrupted tight junction proteins (ZO-1, occludin, and claudin-3) were restored in ETEC K99-infected IPEC-J2 through phage EK99P-1 treatment (Fig. 2A), as evidenced by the preservation of the outer line of ZO-1 expression on ETEC K99 treated IPEC-J2 by EK99P-1 (Fig. 2B). These results suggest that phage EK99P-1 treatment effectively prevents intestinal barrier disruption through inducing increased tight junction proteins in IPEC-J2 infected with ETEC K99.

### Phage EK99P-1 prevented ETEC K99 adherence to IPEC-J2

**Figure 3:**
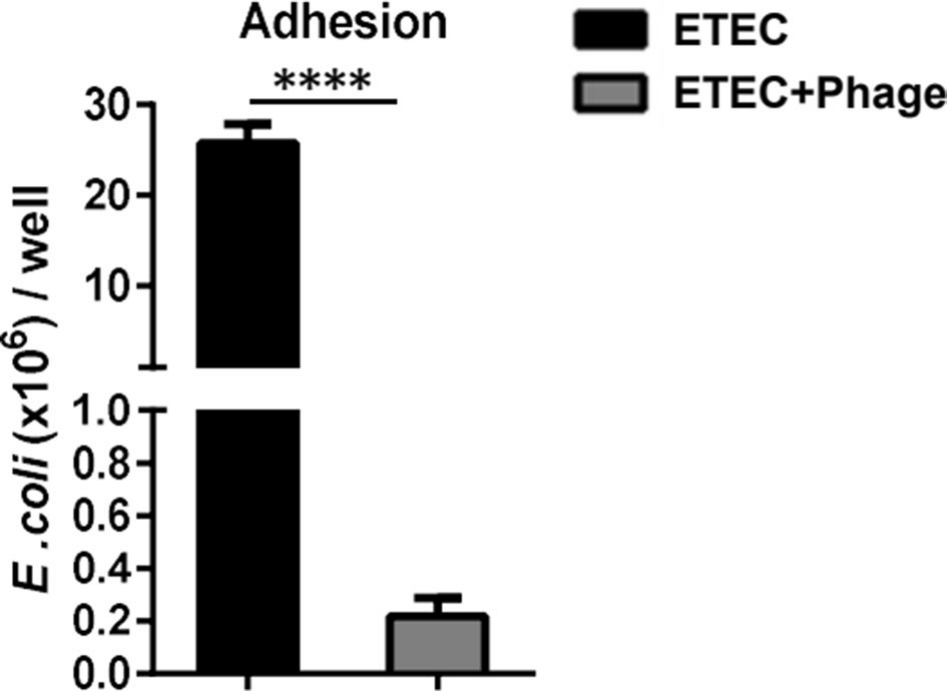
Phage EK99P-1 inhibited the adhesion of ETEC K99 to IPEC-J2. Differentiated IPEC-J2 was treated with ETEC K99 (1 × 10^7^ cfu/mL) and phage EK99P-1 (1 × 10^6^ pfu/mL) for 24 h. An *E. coli* adhesion assay was performed on IPEC-J2 (n = 3). Data are means ± SD. Means were compared using a two-tailed Student’s *t*-test. ****P < 0.0001 compared to the ETEC control.

ETEC adhesion to IECs during infection plays an important role in inducing inflammatory responses and disrupting barrier function at the intestinal lining^8^. Therefore, we hypothesized that the restoration of permeability by phage EK99P-1 in ETEC K99-infected IPEC-J2 could be related to the initial adhesion of ETEC K99 to IPEC-J2. To examine the interference of phage EK99P-1 in the attachment of ETEC K99 to IPEC-J2, we measured the colony-forming units (cfu) of cell-adhesive ETEC K99. We found that phage EK99P-1 treatment reduced ETEC K99 adhesion to IPEC-J2 (Fig. 3). We observed no ETEC K99 translocation from the apical to the basolateral side, with or without phage EK99P-1 (Fig. 2). This result suggests that the intestinal barrier protection conferred by phage EK99P-1 treatment may have begun with reduced ETEC K99 adhesion to IPEC-J2 due to the interference of phage EK99P-1.

### Phage EK99P-1 alleviated inflammatory responses of IPEC-J2 infected with ETEC K99

**Figure 4:**
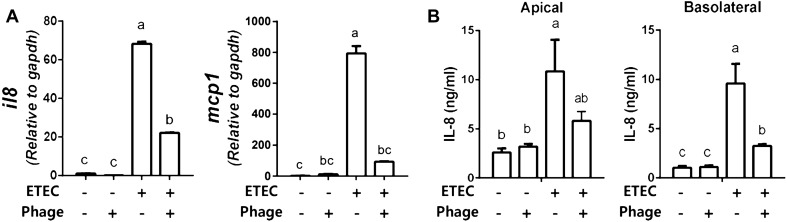
Phage EK99P-1 inhibits inflammatory responses in IPEC-J2 infected with ETEC K99. Differentiated IPEC-J2 were treated with ETEC K99 (1 × 10^7^ cfu/mL) and phage EK99P-1 (1 × 10^6^ pfu/mL) for 24 h. (**A**) The expression of cytokine and chemokine mRNA in differentiated IPEC-J2 was measured by RT-PCR after 24 h of treatment (n = 3). (**B**) IL-8 secretion was measured at supernatant in apical and basolateral side of transwell plate by an enzyme-linked immunosorbent assay (ELISA; n = 3). Data are means ± SD. Means were compared using one-way ANOVA, followed by Friedman’s test and Tukey’s multiple comparison test. Different letters in each group indicate significant differences at *P* < 0.05.

IECs have a barrier function that involves sensing and responding to microbial stimuli, and also participate in the coordination of inflammatory responses. For example, IECs secrete cytokines and chemokines upon recognition of ETEC adhesion^20^ to alert the underlying mucosal immune cells^21^. To investigate whether phage EK99P-1 treatment modulates inflammatory responses following ETEC K99 infection, we examined the release of cytokines and chemokines in IPEC-J2 treated with ETEC K99 and phage EK99P-1. Compared to treatment with ETEC K99 alone, simultaneous treatment of ETEC K99 and phage EK99P-1 significantly reduced the mRNA expression of *IL-8* and *MCP*-1 (Fig. 4A), and significantly decreased IL-8 secretion (Fig. 4B). These results suggest that phage EK99P-1 alleviates inflammatory responses in IPEC-J2 cells infected with ETEC K99.

### Phage EK99P-1 reduced inflammatory cytokines in porcine peripheral blood mononuclear cell (pPBMC) co-cultured with ETEC K99-infected IPEC-J2

**Figure 5:**
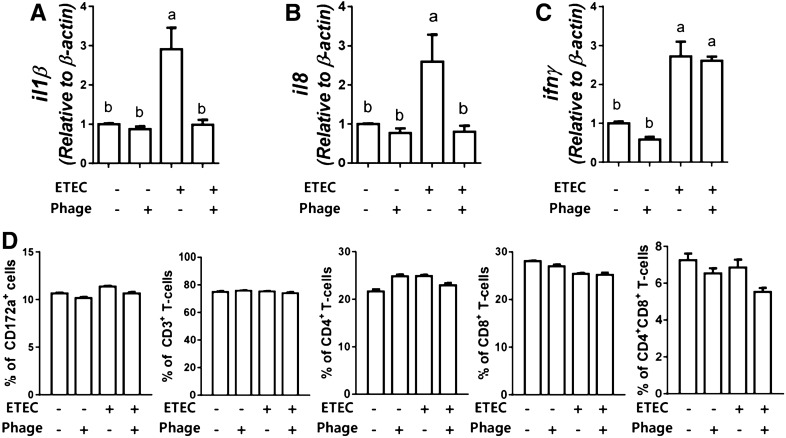
Phage EK99P-1 alleviated the inflammatory response of immune cells under IPEC-J2 infection with ETEC K99. Differentiated IPEC-J2 and pPBMCs were co-cultured using transwell plates. IPEC-J2 were treated with ETEC K99 (1 × 10^7^ cfu/mL) and phage EK99P-1 (1 × 10^6^ pfu/mL) on the apical side for 24 h. (**A–C**) Expression of cytokine mRNA in pPBMC was measured by real-time quantitative RT-PCR (qRT-PCR; n = 3). (**D**) pPBMCs were stained with anti-CD172a, -CD3e, -CD4, or -CD8a antibodies to analyze the population of immune cells using flow cytometry. Data are mean percentages of each cell population relative to the total cells ± SD (n = 3). Means were compared using one-way ANOVA, followed by a Friedman test corrected by Tukey’s multiple comparison test. Different letters in each group indicate significant differences at *P* < 0.05.

IECs are thought to participate in the recognition of bacterial components and transduce signals to resident mucosal immune cells^22^. To investigate the effect of phage EK99P-1 treatment on the modulation of underlying mucosal immune responses by IECs, differentiated IPEC-J2 was co-cultured with pPBMCs using a previously established transwell co-culture system^23^. We investigated the production of pro-inflammatory cytokines and chemokines, *IL-1β*, *IL-8*, and *IFN-γ*, in the co-cultured pPBMCs. The mRNA expression levels of *IL-1β*, *IL-8*, and *IFN-γ* were significantly increased under ETEC K99 alone compared to the non-treated control (Fig. 5A–C). In the co-cultured pPBMCs treated with ETEC K99 in the presence of EK99P-1, *IL-1β* and *IL-8* levels were comparably low to control levels (Fig. 5A,B). However, we observed no differences in the mRNA expression of *IFN-γ* when cells were infected with ETEC K99 in the presence of EK99P-1 compared to ETEC K99 alone (Fig. 5C).

No significant change in the major immune cells composing pPBMCs (CD172a^+^ myeloid cells; CD3^+^ total T cells; or CD4^+^, CD8^+^, or CD4^+^CD8^+^ T cells) was observed following ETEC K99 infection and phage EK99P-1 treatment (Fig. 5D). Together, these data suggest that phage EK99P-1 treatment prevents inflammatory responses caused by ETEC K99 infection by reducing inflammatory cytokines in the immune cells composing pPBMCs.

## Discussion

In this study, we examined the effects of phage EK99P-1 on barrier protection and cytokine production in porcine IECs infected with ETEC in the presence or absence of immune cells. Upon ETEC infection, barrier disruption in the host is thought to occur due to the action of enterotoxins, such as LT or ST, produced from the ETEC. It has been reported that endogenously produced or exogenously added LT substantially enhances the adherence of ETEC to the IPEC-J2^24^. We postulated that the decrease of enterotoxin production caused by phage EK99P-1 inhibits ETEC K99 adhesion to IPEC-J2. As we expected, the attachment of ETEC K99 to IPEC-J2 was decreased when the phage EK99P-1 was added at 24 h after treatment. Bacterial cell adhesion is likely starting at early time point, which might increase in a time-dependent manner in proportion to the bacteria growth. Thus, in our experimental setting, we selected the time that showed the best effect of phages inhibiting bacterial adherence at 24 h. Since enterotoxin gives rise to ETEC pathogenesis by facilitating the attachment of ETEC to host IECs, major barrier disruption is initiated by ETEC adhesion^7,8^. Therefore, the alleviation of barrier disruption by phage EK99P-1 treatment against ETEC K99 infection may be at least partially, if not entirely, caused by decreased ETEC K99 adhesion.

A disrupted barrier favors indiscriminate and easy penetration by pathogens, and impairment of normal physiological function and the local mucosal immune system, often causing an inflammatory reaction^25^. ETEC infection has been reported to induce increased serum IL-8, which was lowered by dietary phage supplementation in post-weaning pigs^12,13^. As previously reported, phage-in-feed reduced the severity of ETEC-induced diarrhea^16–18^, and decreased rectal temperature and *E. coli* adhesion together with a reduction of serum IL-1β, IL-8 and TNF-α^12^ in piglets. ST of ETEC is a key factor inducing IL-8 production in IPEC-J2 cells^26^. We speculated that ST reduction of ETEC K99 by phage K99P-1 could inhibit IL-8 induction in IPEC-J2. Indeed, the current study revealed the induction of IL-8 in cells infected with ETEC K99 in both IECs and immune cells, and its reduction by phage EK99P-1 treatment. Neither IL-1β was significantly increased nor TNF-α was detected (data not shown) after ETEC infection at IPEC-J2 in our experimental setting.

Because IECs are in close contact with immune cells in the lamina propria of intestinal tract, we hypothesized that the modulation of chemokine secretion by phage EK99P-1, in the context of IECs infected with ETEC K99, could influence immune cells at the basolateral side. Therefore, we adopted the IPEC-J2/PBMC co-culture system^23^ and examined the effect of phage EK99P-1 on immune response modulation. Since it is difficult to distinguish the cytokine response between epithelial cells and PBMCs in the co-culture model we have examined mRNA expression by directly sampling PBMCs after the treatment. After co-culture, *IL-1β* and *IL-8* mRNA expression, upregulated by ETEC K99 infection, was diminished by phage EK99P-1 treatment in pPBMCs. One reason for the low levels of inflammatory cytokines in basolateral pPBMCs may be that soluble toxins, such as LT and ST, secreted from ETEC did not permeate the IEC barrier. Our results showed that both 4- and 40-kD dextran-FITC permeability was restored to control levels by phage EK99P-1 treatment against ETEC K99 exposure to IPEC-J2. These results suggest that the basolateral translocation of soluble toxins, especially ST, may also be reduced due to phage EK99P-1 treatment. Indeed, previous reports using enterotoxin-deficient ETEC mutants demonstrated that STb specifically regulates immune-related genes, such as IL-17A, IL-1α, and IL-1β^27^. In addition, ST enhances the expression of pro-inflammatory cytokines and chemokines, such as IL-6 and IL-8, in the small intestine^28^. However, further studies are needed to precisely determine the factors involved in inflammation modulation by immune cells.

Our results also showed that mRNA expression of *IFN-γ*, produced mainly by T cells^29^, was not significantly different in pPBMCs co-cultured with IPEC-J2 infected with ETEC K99, with or without phage EK99P-1. Therefore, it is probable that the control of ETEC K99 by phage EK99P-1 is unrelated to IFN-γ expression by T cells. However, reduced mRNA expression of *IL-1β* and *il8* was observed in the co-cultured system of treated ETEC K99 and phage EK99P-1, compared to ETEC K99 alone. Considering that the main cells producing IL-1*β* and IL-8 in pPBMCs are myeloid cells^30^, we infer a possible difference in cytokine production capacity, although no proportional change in the population of cells was observed. Our results show that phage EK99P-1 blocks intestinal barrier disruption and inhibits inflammatory responses in IPEC-J2 infected with ETEC K99, by reducing ETEC K99 adhesion. Reinforcement of the intestinal barrier by phage EK99P-1 also modulates the immune cell inflammatory response.

## Materials and methods

The use of porcine blood and experimental protocols of the present study were approved by the Institutional Animal Care and Use Committee of Seoul National University and all methods were performed in accordance with the relevant guidelines and regulations.

### Cell culture

IPEC-J2 (DSMZ, Germany), originating from the small intestine jejunum of pigs, were cultured with Dulbecco’s modified Eagle’s medium and F12 Ham’s medium (DMEM-F12; Gibco, USA) containing 5% heat-inactivated fetal bovine serum (FBS; Corning, USA), 1% insulin-transferrin-selenium-X (ITS-X; Gibco), and 1% antibiotics in an incubator at an atmosphere of 5% CO_2_ and 39 °C. For differentiation, IPEC-J2 was seeded at 1 × 10^5^ cells/mL in 500 μL DMEM medium, as described above, on 1.12-cm^2^ polyester membrane inserts (pore size, 0.4 μm); the basolateral side was filled with 1 mL DMEM. After 4 days, the culture medium was replaced with differentiation medium (DMEM-F12 supplemented with 5% heat-inactivated FBS and 1% antibiotics), cultured for 7–10 days, and differentiated within the insert until they attained a TEER of > 5000 Ω.

### Preparation of ETEC K99 and phage EK99P-1

ETEC K99 and phage EK99P-1^31^, that was isolated from the sewage in a swine farm at Asan in South Korea, were donated by iNtRON Biotechnology (Korea). ETEC K99 was spread on an agar plate containing 3% tryptic soy broth (TSB), 1.5% Bacto Agar (all from BD Biosciences, USA) and incubated at 37 °C overnight. One colony was selected for inoculation in 10 mL 3% TSB media, and incubated for 4–5 h in a shaking incubator at 150 rpm and 37 °C until the optical density (OD) reached 1.0. Then, phage EK99P-1 was diluted in elution buffer (10 mM Tris-Cl, pH 8.5) at a density of 7.3 × 10^8^ plaque-forming units (pfu)/mL. The host range having antibacterial activity of phage EK99P-1 is shown in Table 1.

**Table 1:** Host range of bacteriophage EK99P-1 against *E. coli* strain.

Bacterial species	Strain	Plaque formation	Source^a^
*Escherichia coli*	EK99-01^b^	+	1
	EK88-01^c^	−	1
	EF6-01^d^	−	1
	NCCP 13720^e^	+	2
	NCCP 13721^f^	+	2
	ATCC 35150^e^	−	3
	DH5α	−	1
	TOP10	−	1

^a^1: laboratory collection; 2: obtained from the National Culture Collection for Pathogens; 3: purchased from the American Type Culture Collection.

^b^*E. coli* with the F5 (K99) antigen.

^c^*E. coli* with the F4 (K88) antigen.

^d^*E. coli* with the F6 antigen.

^e^Enterohemorrhagic *E. coli* (EHEC) and *E. coli* O157:H7.

^f^Enteroaggregative *E. coli* (EAEC) and enterohemorrhagic *E. coli* (EHEC).

### Bacterial infection and adhesion assay

Differentiated IPEC-J2 cultured in a transwell were washed with pre-warmed phosphate-buffered saline (PBS) and then placed in infection media (DMEM-F12 supplemented with 5% heat-inactivated FBS without antibiotics). The cells were infected with ETEC K99 at a density of 1 × 10^7^ cfu/mL for 24 h at 37 °C and 5% CO_2_. For the adhesion assay, infected IPEC-J2 were washed with pre-warmed PBS three times and then lysed in 0.1% Triton X-100 for 5 min. The lysates were serial diluted and each inoculum, plated onto TSB agar and stored overnight. Viable bacterial cells were quantified as cfu.

### TEER measurement

IPEC-J2 were grown on a 1.12-cm^2^ polyethylene terephthalate membrane insert (pore size, 0.4; Corning, USA) and treated with ETEC K99 and/or phage EK99P-1. TEER was measured using an epithelial volt/ohm meter (EVOM2; World Precision Instruments, USA). Briefly, 0.5 and 1.0 mL of pre-equilibrated medium were added to the apical and basal chambers. Measurements were performed after obtaining a steady signal for 5 min in the blank insert, and corrected by subtracting the background of the blank transwell inserts and medium-only inserts. The final TEER reading was reported in Ω cm^2^ (TEER measurement × area of membrane).

### Dextran permeability measurement

Differentiated IPEC-J2 was cultured in a transwell and treated with ETEC K99 and phage EK99P-1 for 24 h, and then washed with pre-warmed PBS three times. Cell culture medium containing 4 or 40 kDa dextran conjugated with fluorescein isothiocyanate (dextran-FITC) was added to the upper compartment of the transwell plate at 2.2 mg/mL. After 1 h of incubation, the fluorescence intensity in the lower compartment of the transwell plate was measured using a fluorescence multiple plate reader (Victor 3; Perkin Elmer, USA). The excitation and emission wavelengths were 490 and 520 nm, respectively.

### pPBMC isolation

Porcine blood samples were obtained from 4- to 6-month-old Landrace–Yorkshire–Duroc pigs (Hyupsin Food Co., Ltd., Korea). The use of porcine blood was approved by the Institutional Animal Care and Use Committee of Seoul National University (IACUC no. SNU-150327-2). Porcine whole blood was diluted with PBS at a ratio of 1:1, and pPBMCs were isolated by density gradient centrifugation (400×*g* for 20 min without brake) using Ficoll-Paque Plus (Amersham Bioscience, UK)^32^. pPBMCs were suspended in RPMI 1640 medium supplemented with 10% FBS and 1% penicillin/streptomycin.

### Co-culture IPEC-J2/PBMCs

IPEC-J2 was seeded at a density of 1 × 10^5^ cells/mL in 500 μL DMEM medium as described above on 1.12-cm^2^ polyester membrane inserts (pore size, 0.4); the basolateral side was filled with 1 mL DMEM. During cell growth and differentiation, the medium in both compartments was replaced three times per week for 7–9 days. Then, pPBMCs (2 × 10^6^ cells/mL) were seeded in the basolateral compartment of the transwell plate with 1 mL RPMI media, and ETEC K99 and phage EK99P-1 were added to the apical compartment.

### Western blot analysis

To examine the effect of phage EK99P-1 on tight junction proteins, 1 × 10^5^ cells were cultured on 12-well plates for 2 days until confluence was reached. Confluent IPEC-J2 was treated with ETEC K99 and/or phage EK99P-1, washed with PBS and then lysed in a RIPA lysis buffer (50 mM Tris–HCl, 150 mM NaCl, 1% NP_40, 0.5% sodium deoxycholate, 0.1% sodium dodecyl sulfate containing protease inhibitor), followed by protein quantitation using a Micro BCA kit (Thermo, USA). The same amount of extract was loaded into 12% Tris–glycine polyacrylamide gel and electrophoresed. Then, the proteins were transferred onto a polyvinylidene fluoride microporous membrane for 90 min at 4 °C and blocked with 5% skim milk in TBS-T (1 M Tris–HCl, 5 M NaCl, 10% Tween-20) for 1 h. The blot was incubated with rabbit anti-claudin-3, -occludin, and -ZO-1 or mouse anti-β-actin antibodies (Invitrogen, USA) overnight. Then, the membrane was washed and incubated with goat anti-rabbit or anti-mouse IgG-HRP antibody (Santa Cruz Biotechnology, USA) for 1 h. The target protein was visualized using an enhanced chemiluminescence system (GE Healthcare, USA), followed by analysis using a ChemiDoc XRS system (Bio-Rad, USA).

### Confocal immunofluorescence microscopy

IPEC-J2 was treated with or without ETEC K99 in the absence or presence of phage EK99P-1. The cells were washed, fixed with PBS containing 4% formaldehyde (30 min, room temperature), permeabilized with 0.5% Triton-X-100 in PBS for 3 min, and blocked with 10% FBS (30 min, room temperature). Samples were incubated with rabbit anti-ZO-1 antibodies (Invitrogen), followed by staining with goat anti-rabbit IgG conjugated with Alexa Fluor 488 (BD Biosciences, San Jose, USA), and 4′,6-diamidino-2-phenylinodele for nuclei (Immunobioscience, Raleigh, USA). Images was captured using a laser scanning confocal microscope, LSM700 (Carl Zeiss, Jena, Germany).

### Real-time quantitative reverse transcription polymerase chain reaction (qRT-PCR)

Total RNA was isolated using TRIzol reagent according to the manufacturer’s instructions and reverse-transcribed to generate complementary DNA (cDNA) using oligo-dT primers (Bioneer, Korea). Real-time qRT-PCR was performed using a StepOne Plus real-time PCR system (Applied Biosystems, USA). The PCR reaction was conducted in a 96-well reaction plate using 9 μL SYBR green PCR Master Mix, 1 μL each of forward and reverse primers, 1 μL cDNA template, and 8 μL nuclease-free H_2_O. The PCR conditions included 40 thermal cycles of 2 min at 50 °C, 10 min at 95 °C, 15 s at 95 °C, 30 s at 60 °C, and 30 s at 72 °C. Relative quantification of the target genes was calculated using the 2^−ΔΔCt^ method. Target gene expression was normalized to *glyceraldehyde 3-phosphate dehydrogenase* (*GAPDH)* or *β-actin* mRNA level. Primer sequences used in this experiment are shown in Table 2.

**Table 2:** Sequence of oligonucleotide primers used for qRT-PCR.

Target	Primer	Sequence (5′ to 3′)	Size, bp
IL-8	Forward	TGGCAGTTTTCCTGCTTTCT	154
	Reverse	CAGTGGGGTCCACTCTCAAT	
IL-1β	Forward	GTGCAAACTCCAGGACAAAGACCA	120
	Reverse	CACAAGCTCATGCAGAACACCAC	
IFN-γ	Forward	TGGATGTGATCAAGCAAGAC	120
	Reverse	TGGCTTTGCGCTGGATCT	
MCP-1	Forward	AAGTGGGCACACCCGTTTC	120
	Reverse	CGCCATTATGCGTGATTGTT	
GAPDH	Forward	GTCGGTTGTGGATCTGACCT	210
	Reverse	AGCTTGACGAAGTGGTCGTT	
β-actin	Forward	GATGAGATTGGCATGGCTTT	122
	Reverse	CACCTTCACCGTTCCAGTTT	

### Proportional changes of immune cells

We washed pPBMCs with PBS containing 1% FBS and performed staining using the following monoclonal antibodies at pre-determined optimal concentrations: mouse anti-porcine CD3e (clone PPT3; Southern Biotech, USA), CD4 FITC (clone 74-12-4; BD Biosciences), CD8a PE (clone 76-2-11; BD Biosciences), and CD172a biotin (clone 74-22-15; BD Biosciences). Rat anti-mouse IgG1 APC (clone A85-1, BD Biosciences) and streptavidin BV605 (Biolegend, USA) were used as the secondary antibody. The cells were incubated for 20 min at 4 °C in the dark. After staining, the cells were washed and the expression of surface markers was measured using flow cytometry (FACSCanto II; BD Biosciences). All flow cytometric data were analyzed using the FlowJo software (Tree Star, USA).

### Apoptosis analysis

Floating cells were collected and the attached cells were trypsinized for 5 min and washed with PBS. Finally, both trypsinized and floating cells were added together and stained with Annexin V-APC and propidium iodide (PI). Marker intensity was examined using flow cytometry (FACSCanto II; BD Biosciences). All flow cytometry data were analyzed using the FlowJo software (Tree Star).

### Statistical analyses

Means ± standard deviation were determined on the basis of at least three different samples. All experiments were performed at least three times. Means were compared between two groups using two-tailed paired Student’s *t*-tests. The groups were compared by one-way analysis of variance (ANOVA), followed by the Friedman test and Tukey’s multiple comparison test. All statistical analyses were performed using GraphPad Prism software (v5.01; GraphPad Software, USA). Significance was evaluated at a level of *P* < 0.05.

### Ethics declarations

The porcine blood samples used in this study were approved by the Institutional Animal Care and Use Committee of Seoul National University (IACUC No. SNU-150327-2).

## Supplementary Information

Supplementary Figures.
