# Supplementary material for: Bacteriophage EK99P-1 alleviates enterotoxigenic Escherichia coli K99-induced barrier dysfunction and inflammation
Source: Sci Rep. 2022 Jan 18;12:941. doi: 10.1038/s41598-022-04861-4 (PMC8766502; doi:10.1038/s41598-022-04861-4)
Supplement: Supplementary file 1 — Supplementary Figures. [file 41598_2022_4861_MOESM1_ESM.pdf]

Supplementary figure 1. The percentage of necrotic, late apoptotic, early apoptotic cells of IPEC-J2 cells.

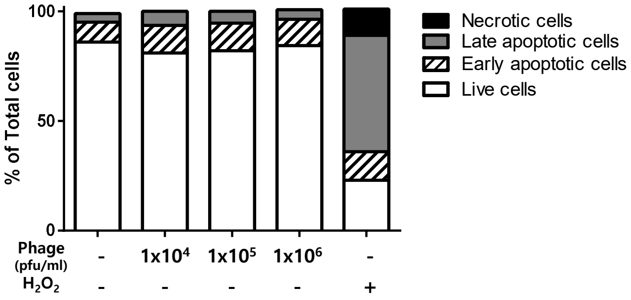

Supplementary figure 1. Monolayer of differentiated IPEC-J2 cell was treated with *E. coli* bacteriophage for 24 h. Then, the cells were stained with anti-AnnexinV and PI, and analyzed for the cell death using flow cytometry. The data represent means of the percentage of cells out of total cells ± SD (n = 3).

Supplementary figure 2. The number of ETEC K99 at the apical and basolateral side of trans-well.

A

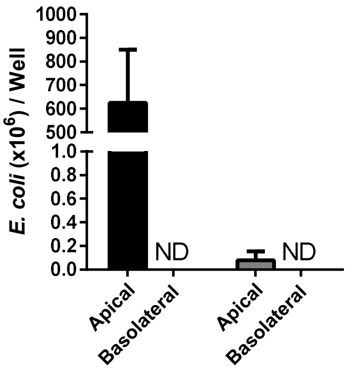

Supplementary figure 2. Differentiated IPEC-J2 cells were treated with ETEC K99 (1×10^7 cfu/ml) and *E. coli* bacteriophage (1×10^6 pfu/ml) for 24 h. Agar plating assay show (A) the number of *E. coli* on supernatant from apical and basolateral side (n = 3). ND denotes not detected.

Supplementary figure 3. The percentage of necrotic, late apoptotic, early apoptotic cells of pPBMC.

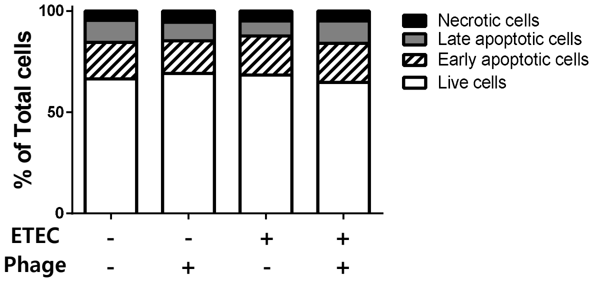

Supplementary figure 3. Differentiated IPEC-J2 cells and pPBMCs were co-cultured using trans-well plate. IPEC-J2 cells were treated with ETEC K99 (1×10^7 cfu/ml) and *E. coli* bacteriophage (1×10^6 pfu/ml) to the apical side for 24 h. (A) Then, the pPBMCs were stained with anti-AnnexinV and PI and analyzed using flow cytometry. The data represent means of the percentage of cells out of total cells ± SD (n = 3).

Supplementary figure 4. Full-length blot of figure 2

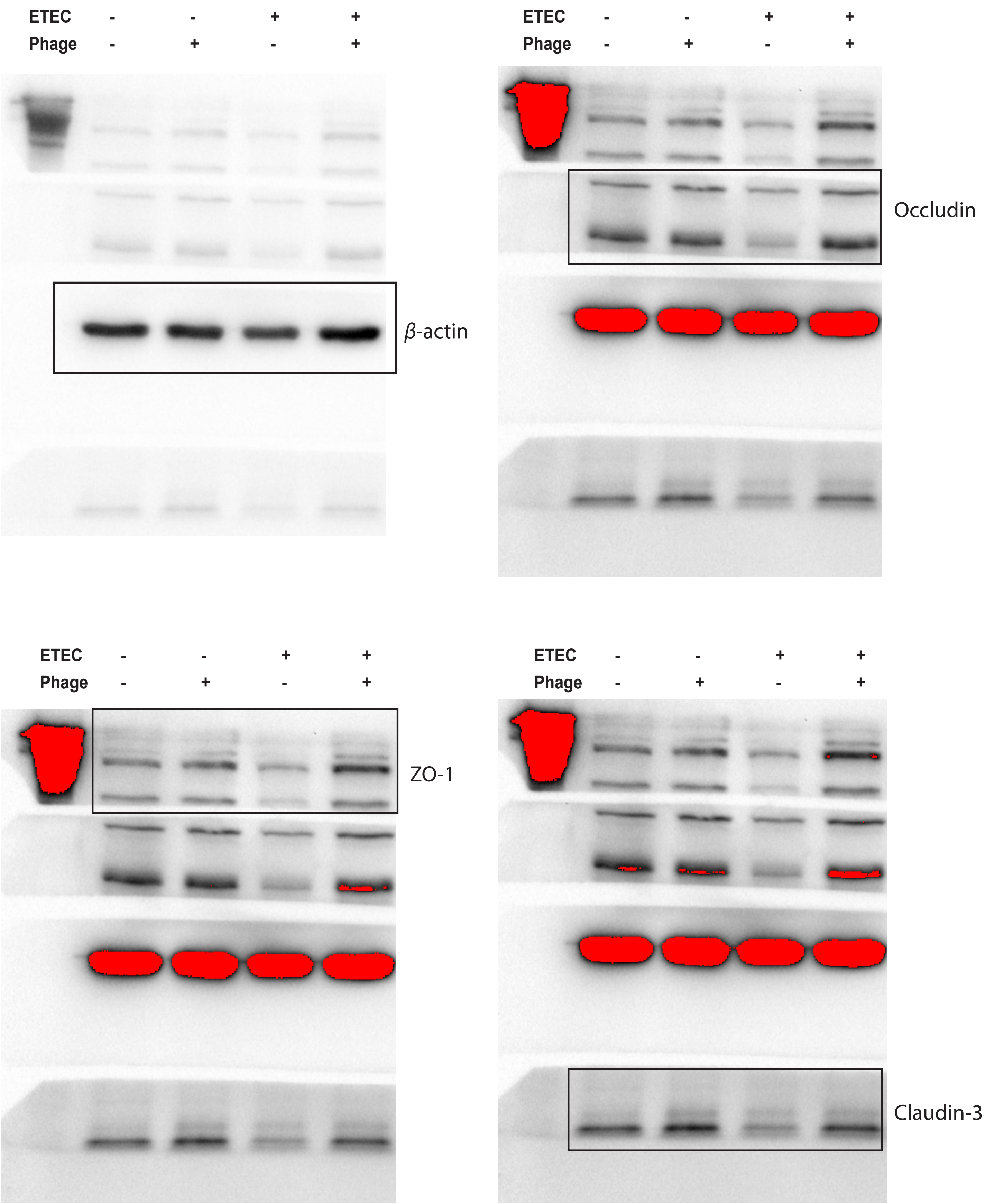

Supplementary figure 4. Each image represents the same gel with different exposure times, and image in the black box is used for the original figure 2.
